# Association between physical limitations and depressive symptoms among Indian elderly: marital status as a moderator

**DOI:** 10.1186/s12888-021-03587-3

**Published:** 2021-11-16

**Authors:** Babul Hossain, Pawan Kumar Yadav, Varsha P. Nagargoje, K. J. Vinod Joseph

**Affiliations:** 1 International Institute for Population Sciences, Mumbai, 400088 India; 2 Department of Bio-statistics and Epidemiology, International Institute for Population Sciences, Mumbai, 400088 India

**Keywords:** Elderly depressive symptoms, Physical limitations, ADLs, IADLs, Mobility difficulty, Marital status

## Abstract

**Background:**

Depression among the elderly is well-documented and associated with socio-economic factors, physical and mental health conditions. Few studies have focused on older adults’ physical limitations and depressive symptoms. However, very little is known about marital status’ role in such associations, especially in India. The present study examines the association between physical limitations and self-reported depressive symptoms and moderating role of marital status in such association separately for men and women.

**Methods:**

The present study used data from the Longitudinal Ageing Study in India (LASI) wave 1, 2017–2018, a nationally and state representative longitudinal large-scale survey of ageing and health. For the present research, a total sample of 20,806 older adults aged 60+ years was selected after excluding missing values. Along with descriptive statistics, binary logistic regression analysis and interaction effect of marital status were applied to examine the association between physical limitations (functional limitations and mobility difficulty) with the depressive symptoms separately for men and women.

**Results:**

About 58, 50, and 45% elderly reported having depressive symptoms and had difficulty in 2+ ADLs, 2+ IADLs, and 2+ mobility difficulties, respectively. By the marital status, the prevalence of depressive symptoms was higher among currently unmarried than currently married, irrespective of type and number of physical limitations. The unadjusted, marital and multivariate-adjusted association suggested that elderly with more than two ADLs, IADLs, and mobility difficulty had higher odds of depressive symptoms. The gender stratified interaction effect of marital status and physical limitations on depressive symptoms indicated that currently unmarried elderly, particularly unmarried older women with 2+ ADLs (OR = 2.85; CI 95% = 1.88–3.09), 2+ IADLs (OR = 2.01; CI 95% = 1.74–2.31) and 2+ mobility difficulty (OR = 2.20; CI 95% = 1.86–2.60) had higher odds of depressive symptoms. However, such association was only valid for unmarried men having mobility difficulty.

**Conclusion:**

The study highlights that the elderly with physical limitations such as ADLs, IADLs, and mobility difficulty require attention and care. Although married elderly are less likely to have depressive symptoms even with all the mentioned physical limitations, unmarried women are more vulnerable to have depressive symptoms with physical limitations.

## Background

Geriatric depression is an emerging public health concern and a greater social challenge. According to various studies, the prevalence of depression among middle-aged and older people ranges from 1 to 16% worldwide [1–3]. Depression intensifies complications in treating physical illness, increases the risk of emerging new diseases, and elevates mortality [4, 5]. Generally, elderly depression is considered the outcome of poor physical and mental health conditions and socio-economic difficulty [1, 6–11].

A substantial amount of research conducted in developed countries has suggested a strong relationship between functional impairment and levels of depressive symptoms in older individuals [12–15]. Evidence suggests limitations in instrumental activities of daily livings (IADLs), such as shopping, meal preparation, and housekeeping, and personal activities of daily livings (ADLs), viz. bathing, eating, toileting, and dressing, have been associated with greater levels of psychological distress [16]. Physical limitations can accelerate the individuals’ depression level for several reasons. Functional limitations or obstacles in mobility lead to compromise with the number of day-to-day activities in an individual’s life. It has been found that the increase in physical limitations directly influences an individual’s professional and personal life [17–20]. As a result, the physical limitations have negatively influenced participation in social activity and affect subjective health and psychological well-being [21–26]. With such physical limitations, individuals become dependent on others for minimal tasks and start to feel less control over their bodies and activities, stimulating psychological distress [27]. However, some evidence also suggested the bidirectional relationship between physical limitations and depressive symptoms. In that context, Aneshensel and colleagues’ (1984) conducted one of the first investigations to evaluate such reciprocal associations [13]. A study based on community-level adult samples found that physical illness and depression exert reciprocal effects over time. More recently, additional studies have also appeared to confirm such a reciprocal relationship [14]. These studies considered the temporal and reciprocal associations between physical limitations and depressive symptoms. However, in his study, Gayman et al. (2008) indicated that prior levels of physical limitations predicted changes in depressive symptoms but did not find the reverse association [19].

Previous studies have shown a significant association between geriatric depression and functional limitations with several socio-demographic, economic, and health -related factors [1, 6–9, 28, 29]. However, some scholars argued that marital status as a social determinant of health is crucial in such a relationship [12, 30]. The marital protection hypothesis suggested that individuals get better attention and care within the marital union, even prevention from the effects of physical limitations [31–33]. The spouse is the primary source of support and caregiving and assists their marital partner with physical limitations [34]. On the other hand, studies also suggest positive implications of marriage by increasing subjective well-being, reducing loneliness, anger, chronic depressive symptoms, and stress [35–38]. Some studies have also explored whether marital status alters the association between different factors and psychological distress. For instance, Gove’s (1972), in his sex-role theory of mental illness, attributes female preponderance of psychological distress to their roles in contemporary U.S. society [39]. Studies also found that the husband showed less psychological symptoms than their wives due to lower interpersonal connections [40, 41].

Although limited studies have analysed the moderating effect of marriage on the association between physical limitations and psychological distress, studies indicate that marriage tends to moderate the direction and magnitude of the relationship of physical limitations with depression and other psychological distress. For example, Bierman (2012) reported that marriage decreased the relationship between functional limitations and depression, only significant for older men [12]. Han et al. (2021) examined the association of activity limitations with depressive symptoms moderated by receiving and providing spousal care and found that persistent moderating spousal care influenced the link between one’s own activity limitations and depressive symptoms [42]. Focusing on the moderating effect of spousal support, Marini et al. (2020) indicated that spousal support was an essential factor in reducing depression among functionally limited individuals and correlated with lower levels of loneliness [43]. However, Marini and the team conclude that poor marriage quality, including marital conflict, can increase depressive symptoms via functional impairment [43].

The association between marital status and health outcome is persisting but significantly varies with gender [12, 42, 44, 45]. However, findings are inconsistent and vary across countries and study populations. The role theory of marriage suggests that although women in a marital union get economic advantages, marriage is more socially and physiologically beneficial for men than women [44, 46]. A study also reported that married men with functional limitations had lower depressive symptoms as they received care and assistance from their wives. However, such a relation was missing for the women [43]. In contrast, shreds of evidence also suggested that differential marital status significantly affects women’s psychological state than men’s [47]. At the same time, some studies did not find any gender gap in mental health due to marital dissolution [48].

As per census 2011, the share of the Indian older persons (60 years and above) comprises 8.6% of the total population in India [49]. This proportion is expected to reach around 19% by 2050 [50]. Furthermore, one in every seven older adults in India has difficulty performing at least one physical and instrumental activity of daily living [51]. Along with this, one-third of the total aged population suffering from depression prompts the need for research on psychological distress among Indian aged people [52]. Around 34.4% of older Indian individuals exhibited depressive symptoms from 1997 to 2016, comparatively higher than reported for most Asian and Western countries of the world [52].

Numerous studies on the Indian population have investigated physical health, functional limitations, and psychological health and association with different factors and marital status, suggesting that marriage is positively associated with better health, lower depressive symptoms, and less physical limitation than the unmarried persons [31, 53, 54]. For instance, studies found that married elderly had a lesser risk of mobility difficulty whereas unmarried status was disadvantageous, particularly for the women [55, 56]. Whereas recently, widowed women are more likely to suffer from distress [53, 57]. However, the existing pieces of literature are not enough to depict the scenario for entire India. Moreover, there has been no study focusing on the association of physical limitations with depressive symptoms and considering the role of marital status despite knowing marriage provides a physical and mental health advantages to older individuals.

The present study has two primary aims: i) to examine the association between physical limitations and depressive symptoms and ii) to examine the role of marriage in moderating the association between physical limitations and depressive symptoms separately for men and women.

## Material and methods

### Data source

The present research used data from the Longitudinal Ageing Study in India (LASI) wave 1, 2017–2018, a nationally and state representative longitudinal large-scale survey of ageing and health, particularly for older adults aged 45 and above and their spouses irrespective of their ages. LASI provides valid, reliable, and continuous scientific data on a targeted population’s health, social, mental, and economic well-being. The targeted sample comprises non-institutionalised Indian residents chosen through the multistage stratified area probability cluster sampling design from all 30 states (excluding Sikkim) and six Union Territories of India [58].

LASI adopted a three-stage sample design in rural areas and a four-stage sample design in urban areas. At the first stage, as per the 2011 Indian census, the list of sub-districts (Tehsils/Talukas) was considered as Primary Sampling Units (PSUs) for each state/UT. In each region, the PSUs were selected using Probability Proportional to Size (PPS) sampling with the number of households in a PSU as the size measure. The second stage involved selecting a fixed number of Secondary Sampling Units (SSUs), which are villages from rural areas and wards from urban areas of the selected PSUs. A fixed number of households were chosen from selected villages using systematic sampling at the third stage in rural areas. However, sampling in urban areas involved one more stage. In the third stage, one Census Enumeration Block (CEB) was randomly selected from each selected urban ward. At the fourth stage, a fixed number of households from this CEB were systematically selected. The goal of this extensive sampling framework was to choose a representative sample in each stage of sample selection. Further, an individual survey schedule was administered to each consenting respondent aged 45 and above and their spouses (irrespective of age) in each sampled household. Detailed information about the sampling framework and selection of sample size is available in the national report of LASI, wave 1, 2017–18, India [58].

### Study sample

**Fig. 1:**
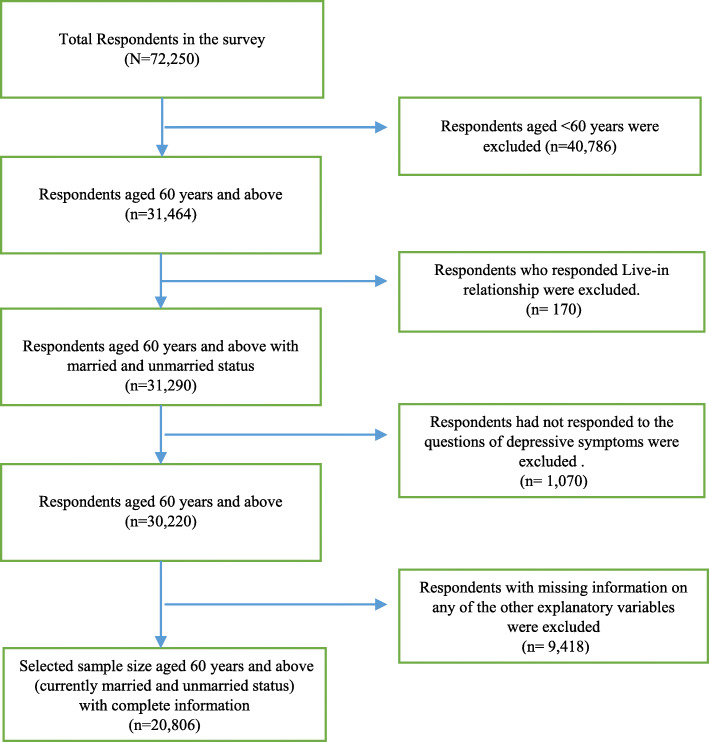
A flow chart describing sample selection for the study, LASI wave 1, 2017–18

LASI wave 1 provided information on the total sample of 72,250 aged 18 and above and their spouses irrespective of their ages without any missing value in age reporting. Our study population was older adults aged 60 and above with currently married and currently unmarried status. Thus below 60 years sample were droped (*n = 40,786*). Unmarried respondents’ category includes those who were reported their current marital status as widowed/ divorced/ separated/ deserted or never married. In the Indian context, *live-in-relationship (n = 170)* is not considered as ‘married’; hence we had dropped the sample under the category of live-in-relationship. So, the sample size reduced up to 31,290. The dependent variable was depressive symptoms among the older adults. Respondents who had not responded to the questions of depressive symptoms (*n = 1070*) were considered missing values. Also, information on any of the other explanatory variables such as work status, caste, ADL/IADL, self-rrated health, household size, etc., also consist of missing values that we have dropped. Hence, after dropping the sample containing missing values, the final sample became 20,806 which consisted of 13,132 men and 7674 women. Figure 1 provides a flowchart of the final sample selection for the study.

### Variable description

#### Outcome variable

##### Depressive symptoms

The outcome variable was a binary classification of depressive symptoms. In the LASI survey, the reference period for the depressive symptom was considered of past 1 week from the survey date. In the individual schedule, a question has been asked to the respondents “How often did you feel depressed?” which had responses of “rarely or never (less than one day)”, “Sometimes (one or two days)”, “Often (three or four days)”, and “Most or all of the time (five to seven days)”. It was then recorded “0” for no self-reported depressive symptoms for the response with “rarely or never”, and the rest of the responses were categorised as “1” for depressive symptoms [59].

#### Explanatory variables

##### Physical limitations

The physical limitations in the study meant functional limitations consisting of personal Activities of Daily Livings (ADLs) and Instrumental Activities of Daily Livings (IADLs) and mobility difficulty. ADLs consist of six normal daily self-care activities related to difficulties, such as difficulty with dressing, including putting on chappals or shoes, walking across a room, bathing, eating, getting in or out of bed, using the toilet, including getting up and down. Combining together these six ADLs, a single variable was generated and was recorded as “no ADL” if the respondent did not face difficulty in performing any ADL, “1 ADL”, if the respondent had difficulty in performing only one ADL and “2+ ADLs” if the respondent had difficulty in performing more than 2 ADLs. Further, IADLs comprised of seven instrumental activities related difficulties which performed regularly. For instance, preparing a hot meal (cooking and serving), shopping for groceries, making telephone calls, taking medications, doing work around the house or garden, managing money such as paying bills and keeping track of expenses, getting around or finding the address in unfamiliar place were considered to measure Instrumental Activities of Daily Livings (IADLs). Similar to ADLs, IADLs was recorded as “no IADL”, “1 IADL” and “2+ IADLs”. Nine task-related difficulties in mobility such as walking 100 yards, sitting for 2 h or more, getting up from a chair after sitting for a long period, climbing one flight of stairs without resting, stooping, kneeling or crouching, reaching or extending arms above shoulder level (either arm), pulling or pushing large objects, lifting or carrying weights over 5 k, like a heavy bag of groceries, picking up a coin from a table were considered to measure mobility difficulty. Mobility difficulty was also categorised as “no mobility difficulty”, “1 mobility difficulty” and “2+ mobility difficulty”.

#### Marital status

Existing studies have categorised marital status into different categories such as single, married, widowed, divorced, and separated [41]. However, our study only aims at marriage and its role in the association between physical limitations and depressive symptoms and does not focus on other nonmarried categories despite knowing that the relationship across different marital categories may vary. Thus, for the current study, marital status was categorised as currently married and currently unmarried (including widowed, divorced, separated, deserted and never married).

### Covariates

Other covariates considered and controlled in the analysis were the socio-demographic, economic, and health-related factors like age (60–69, 70–79 and 80+), education (ever attended school and never attended school), place of residence (rural and urban), income (poorest, poorer, middle, richer, richest), working status (yes and no), caste (SC, ST, OBC and Others), number of household members (1–2, 3–4, 5–6, 7 or more), and self-rated health (good and bad).

### Statistical analysis

Binary logistic regression analysis was used to examine the association between functional limitations and mobility difficulty with the symptoms of depression. The gender-wise interaction effects of the functional limitations and marital status on the symptoms of depression in older adults aged 60 and above were examined using adjusted and unadjusted binary logistic regression analysis. Depressive symptoms had two categories; it takes the value of 1 for self-reported depressive symptoms and 0 for no self-reported depressive symptoms at all.

The equation of the logistic regression is as follows:

$$ \mathrm{Logit}\ \left(\mathrm{Y}\right)=\ln\ \left(\mathrm{p}/\left(1-\mathrm{p}\right)\right)=\alpha +{\beta}_1{X}_1+{\beta}_2{X}_2+{\beta}_3{X}_3+\dots \dots .+{\beta}_k{X}_k+\in $$

Here, *β*_1_, *β*_2_, *β*_3_ … … . *β*_*k*_ were the regression coefficients and showed the relative effect of a particular socio-demographic factor on the outcome variable, and the coefficients change according to the context of the analysis. All the statistical analysis is done using STATA (version 16) and MS excel program.

## Results

Table 1 shows the percentage of self-reported depressive symptoms, physical limitations, and socio-economic profile of older adults (aged 60 years and above) in India. Around 42% of the individuals were reported with depressive symptoms, and among them, 39% were men, and 47% were women. Compared to men (12%), more women (17%) could perform more than two ADLs. The percentage of two or more than two IADLs was 34% in the total sample. However, it was nearly double among the women (47%) than the men (26%). An average of 64% of the older adults had more than two mobility difficulties. However, such proportion was 14% higher among the older women (73%) than their men counterparts (59%). Above 65% sample was in 60 to 69 years of age group. More than three-fifth of older men and only around one-fifth of older women had ever attended the school. Looking at the marital status, the proportion of currently married older men was almost double that of their female counterparts. A higher proportion of older men were currently working. About 48% of older adults reported poor self-rated health.

**Table 1:** Characteristics of the study population, LASI wave 1, 2017–18

		Total sample (***N*** = 20,806)	Men (***N*** = 13,132)	Women (***N*** = 7674)
**Characteristics**	**Category**	**Percentage**		
**Self-reported depressive symptoms**	No	58.32	61.35	53.45
	Yes	41.68	38.65	46.55
**ADL**^**#**^	No	78.09	80.53	74.16
	1	7.97	7.20	9.22
	2+	13.94	12.27	16.62
**IADL**^**##**^	No	54.56	62.26	42.16
	1	11.55	11.74	11.26
	2+	33.89	26.00	46.58
**Mobility difficulty**	No	26.63	31.00	19.61
	1	9.14	10.22	7.41
	2+	64.22	58.78	72.98
**Age**	60–69	68.01	67.08	69.50
	70–79	24.69	25.32	23.66
	80+	7.31	7.60	6.84
**Education**	Ever attended school	45.20	61.76	18.55
	Never attended school	54.80	38.24	81.45
**Place of residence**	Rural	70.50	67.60	75.30
	Urban	29.50	32.70	24.60
**Marital status**	Currently married	66.85	81.80	42.79
	Currently unmarried^*^	33.15	18.20	57.21
**Income**	Poorest	22.57	21.19	24.77
	Poorer	21.90	21.73	22.17
	Middle	21.07	21.01	21.15
	Richer	18.94	19.08	18.72
	Richest	15.52	16.98	13.19
**Working status**	Yes	42.94	46.90	36.58
	No	57.06	53.10	63.42
**Caste**	Scheduled Tribe (ST)	10.07	8.54	12.53
	Scheduled Caste (SC)	21.29	20.03	23.31
	Other Backward Class (OBC)	46.07	45.85	46.42
	Others	22.58	25.58	17.74
**Number of household members**	1–2 members	25.83	23.22	30.03
	3–4 members	20.55	21.93	18.34
	5–6 members	28.38	27.60	29.63
	7 or more members	25.24	27.25	22.00
**Self-rated health (SRH)**	Good self-rated health	52.25	53.52	50.20
	Poor self-rated health	47.75	46.48	49.80

***Note:*** *Individual sampling weights given in LASI wave 1, 2017–18 are applied*

^*#*^ *Activities of Daily Living (ADL)*, ^*##*^ *Instrumental Activities of Daily Living (IADL)*

*Currently unmarried category comprises widowed, divorced, separated, deserted and never married older individuals

**Fig. 2:**
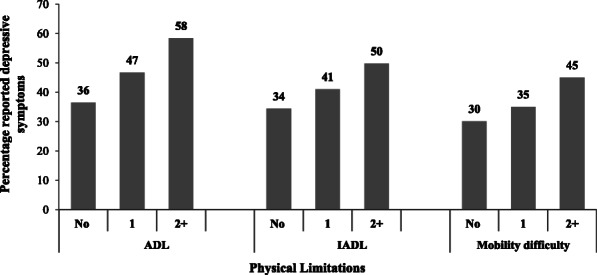
Percentage of elderly self-reported depressive symptoms by physical limitations, LASI wave 1, 2017–18

Fig. 2 shows the percentage share of elderly depressive symptoms by physical limitations in India. With the increased number of tasks difficulties, whether for ADLs, IADLs, or morbidity difficulty, symptoms of depression also increased. The percentage of older adults’ depressive symptoms was 58% among persons having difficulty with two or more ADLs, followed by 47% among persons with difficulty with one ADL. The lowest depressed individuals were with no difficulty in ADL (36%). Similarly, the elderly depressive symptoms were highest among persons facing difficulty with two or more IADLs (50%), followed by those with difficulty with one IADL (41%) and lowest among persons with no difficulty in IADL in India. A similar pattern was also observed for the elderly facing mobility difficulty.

**Fig. 3:**
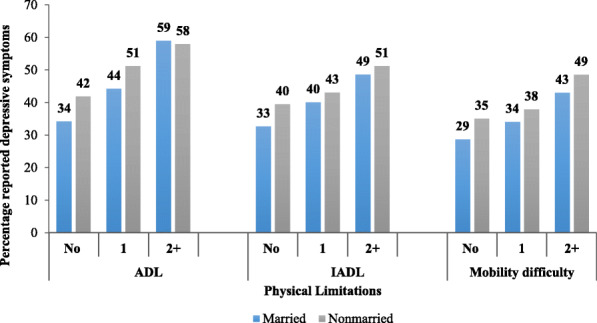
Prevalence of elderly self-reported depressive symptoms by physical limitations and marital status, LASI wave 1, 2017–18

The prevalence of self-reported depressive symptoms among the elderly by physical limitations and marital status has given in Fig. 3. The results showed a visible variation in depressive symptoms among currently married and unmarried older adults across all physical limitation categories. The prevalence of depressive symptoms was lowest among the aged people who were not facing ADL, IADL, and mobility difficulties and highest among those with 2+ ADL, 2+ IADL, and 2+ mobility difficulty issues. Among which 34.2% of currently married individuals without any issues of ADL reported depressive symptoms, while it was 42% among currently unmarried individuals without any issues of ADL. About 33% of married individuals without any issues of IADL reported depressive symptoms, while it was 40% among currently unmarried individuals without any issues of IADL. The depressive symptoms levels were highest among currently unmarried elderly (51.2%) than currently married counterparts (48.6%) with 2+ issues of IADL. When the mobility difficulty was considered, the levels of depressive symptoms was lowest among currently married (28.7%) and currently unmarried (35.1%) elderly without any mobility issues and highest among unmarried (48.6%) elderly than married (43%) with 2+ mobility issues.

Table 2 shows the association between physical limitations with self-reported depressive symptoms in India. Multivariate logistic regression analysis (unadjusted, marital status adjusted, and multivariate-adjusted) suggested a significant positive association between functional limitations and mobility difficulty with self-reported depressive symptoms. The unadjusted association in model 1 shows that elderly having one ADL difficulty were 1.5 times, and two or more than two ADLs difficulty were 2.4 times more likely to have depressive symptoms than the elderly with no ADL difficulties. The elderly with 1 IADL difficulty were 1.3 times more likely, and 2+ IADL difficulty were 1.8 times more likely to have depressive symptoms than the reference category. The unadjusted result for mobility difficulty was also almost similar to IADLs. Marital adjusted and multivariate-adjusted results also indicated significant positive associations between physical limitations and depressive symptoms among the elderly population. However, the association’s magnitude had slightly decreased compared to the unadjusted association.

**Table 2:** Association between physical limitations with self-reported depressive symptoms among the Indian older adults, LASI wave 1, 2017–18

Main variable	Model 1 OR (95% CI)	Model 2 OR (95% CI)	Model 3 OR (95% CI)
**ADL**^**#**^			
No ADL (ref)			
1 ADL	1.52***(1.37 1.70)	1.50***(1.35 1.67)	1.43***(1.28 1.59)
2+ ADL	2.44***(2.24 2.66)	2.38***(2.18 2.59)	2.19***(2.00 2.39)
**IADL**^**##**^			
No IADL (ref)			
1 IADL	1.32***(1.21 1.45)	1.31***(1.19 1.43)	1.24***(1.13 1.36)
2+ IADL	1.89***(1.77 2.01)	1.81***(1.70 1.93)	1.67***(1.56 1.788)
**Mobility difficulty**			
No mobility difficulty (ref)			
1 mobility difficulty	1.25***(1.12 1.39)	1.24***(1.11 1.38)	1.20***(1.07 1.34)
2+ mobility difficulty	1.89***(1.77 2.02)	1.84***(1.72 1.96)	1.66***(1.55 1.79)

***Note:*** *OR: Odds Ratio*, ^*#*^*Activities of Daily Living (ADL),* ^*##*^ *Instrumental Activities of Daily Living (IADL) ref: Reference category, Model 1 is the unadjusted, Model 2 is marital status adjusted and Model 3 is all factor adjusted association of functional limitations and mobility with symptoms of depression*

Table 3 shows the gender stratified interaction effect of the physical limitations and marital status on Indian older adults’ depressive symptoms. The adjusted and unadjusted logistic regression analysis with the interaction effect of physical limitations and marital status on depressive symptoms shows a significant positive association in both men and women. Even without any physical limitation, unmarried had higher odds of depressive symptoms than currently married. However, in such conditions, the magnitude was higher for currently unmarried women than unmarried men. Women who were currently unmarried with 2 + ADLs had higher odds of depressive symptoms (OR = 2.85; CI 95% = 1.88–3.09) than those who had no ADL and were currently married. However, married women with 2+ ADLs also had significantly higher odds of depressive symptoms than any group. Unmarried women with 2+ IADL were 2.1 times more likely, followed by currently married women with 2 + IADL, 1.8 times more likely to have depressive symptoms with reference to the currently married women with no IADL difficulties.

**Table 3:** Gender stratified interaction effect of physical limitations and marital status on the self-reported depressive symptoms in Indian older adults, LASI wave 1, 2017–18

	Women		Men	
Main variable	Unadjusted (OR)	Adjusted^**#**^ (OR)	Unadjusted (OR)	Adjusted^**#**^ (OR)
**ADL**^**##**^ **and marital status**				
No ADL * currently married	1	1	1	1
No ADL* currently unmarried	1.43***(1.29 1.59)	1.43***(1.28 1.59)	1.19***(1.07 1.32)	1.17***(1.05 1.30)
1 ADL * currently married	1.81***(1.40 2.34)	1.76***(1.36 2.28)	1.42***(1.22 1.66)	1.35***(1.15 1.58)
1 ADL * currently unmarried	2.23***(1.79 2.78)	2.20***(1.75 2.75)	1.44**(1.05 1.98)	1.34*(0.98 1.85)
2+ ADL * currently married	2.78***(2.24 3.46)	2.60***(2.09 3.25)	2.82***(2.46 3.18)	2.54***(2.22 2.90)
2+ ADL * currently unmarried	2.95**(1.99 3.17)	2.85**(1.88 3.09)	2.81***(2.19 3.56)	2.51***(1.96 3.21)
**IADL**^**###**^ **and marital status**				
No IADL * currently married	1	1	1	1
No IADL * currently unmarried	1.44***(1.25 1.65)	1.43***(1.24 1.65)	1.18***(1.04 1.34)	1.17**(1.03 1.33)
1 IADL * currently married	1.42***(1.14 1.78)	1.37***(1.09 1.71)	1.35***(1.19 1.53)	1.29***(1.14 1.47)
1 IADL * currently unmarried	1.74***(1.42 2.15)	1.67***(1.35 2.07)	1.20 (0.93 1.56)	1.16 (0.89 1.51)
2+ IADL * currently married	1.81***(1.56 2.10)	1.70***(1.46 1.99)	2.00**(1.82 2.19)	1.83***(1.65 2.02)
2+ IADL * currently unmarried	2.11***(1.85 2.40)	2.01***(1.74 2.31)	2.12***(1.81 2.48)	1.97***(1.67 2.32)
**Mobility difficulty and marital status**				
No MD * currently married	1	1	1	1
No MD * currently unmarried	1.42***(1.15 1.75)	1.43***(1.16 1.76)	1.16 (0.96 1.44)	1.43***(1.24 1.65)
1 MD * currently married	1.08 (0.81 1.45)	1.07 (0.80 1.43)	1.33***(1.16 1.53)	1.37***(1.09 1.71)
1 MD * currently unmarried	1.44**(1.09 1.91)	1.41**(1.06 1.87)	1.52***(1.12 2.05)	1.67**(1.35 2.07)
2+ MD * currently married	1.84***(1.56 2.17)	1.72***(1.45 2.05)	1.87***(1.71 2.04)	1.70*(1.46 1.99)
2+ MD * currently unmarried	2.34***(2.00 2.75)	2.20***(1.86 2.60)	2.07***(1.83 2.35)	2.01***(1.74 2.31)

***Note:*** ^**#**^ *Adjusted association include age, education, place of residence, income, working status, caste, HH size and self-rated health*

*OR: Odds Ratio*, ^*##*^ *Activities of Daily Living (ADL),* ^*###*^ *Instrumental Activities of Daily Living (IADL)*

*MD denotes Mobility difficulty*

Similarly, unmarried women with 2+ MD were 2.2 times more likely and currently married women with 2+ MD were 1.7 times more likely to have depressive symptoms than the currently married women who were not facing any mobility difficulty. However, such a difference between married and nonmarried was not found for men with difficulties in ADLs. For example, unmarried men with one or two and above ADLs had almost the same odds ratio of having depressive symptoms as married men. For men with IADLs, the conditions were also identical. The adjusted results of the mobility difficulty and its interaction with marital status show that unmarried men with 2+ mobility difficulties had 2.01 (CI 95% = 1.74–2.31) times more likely, followed by currently married men with 2+ mobility difficulty 1.7 times more likely, to have depressive symptoms with reference to currently married men with no mobility difficulty.

## Discussion

This study has examined the association between physical limitations and depressive symptoms and examined the moderating role of marital status in this association. We have found that with the increase in the number of difficulties in the selected physical limitations, self-reported depressive symptoms level among the elderly also increases and the most remarkable result to emerge from the data is that the prevalence of depressive symptoms is higher among unmarried than married, irrespective of type and number of physical limitations. The correlation between physical limitations and depressive symptoms is worth mentioning because the association, whether unadjusted, marital, or multivariate-adjusted, physical limitation emerges as a strong predictor of depressive symptoms among the elderly population. Our study provides additional support for the marriage support hypothesis in the association between physical limitations and depressive symptoms. We find that unmarried elderly, particularly unmarried older women with physical limitations, have higher odds of depressive symptoms. However, such association is only valid for unmarried older men having mobility difficulty.

Our study found that physical limitation negatively associated with depressive symptoms among older adults in India. These results are consistent with previous research in similar settings [24, 26, 27]. When the elderly experience difficulties performing their daily activities and reducing social interactions, their depressive symptoms increase [60]. Mobility is also identified to affect depressive symptoms levels significantly. The possible explanation could be that individuals started depending on others for everyday tasks and believed that limitation over day-to-day activity further stimulated psychological distress [27]. Fagerström & Borglin, (2010) also found that physical immobility was likely to increase depressive symptoms and further concluded that the life satisfaction of the immobile elderly decreases over time because it was essential for having an independent life and undertaking daily activities, causing elevated depressive symptoms among the immobile elderly [61].

The current research shows a significant moderating role of marriage on the relation between physical limitations on depressive symptoms of the elderly. Both longitudinal and cross-sectional studies have identified marital status as a risk factor for depressive symptoms in the elderly [62, 63]. However, the role theory of marriage suggests that marriage is more socially and psychologically beneficial for men than women, and existing literature found that married males with functional limitations had lower depressive symptoms than the unmarried male with functional limitations. However, such a relation was missing for the women [30, 44, 46]. Contrary to earlier findings, we did not find that marital status is a significant moderating factor influencing the association between ADLs and IADLs with depressive symptoms among men. Whereas, we found a significant moderating role of marital status in the association between physical limitations and depressive symptoms among women, contradicting the results of previous literature, which reported a lower level of depressive symptoms among women irrespective of their marital status [30, 44].

Our results may differ from other study findings because existing studies on moderating role of marriage on the association between depressive symptoms and physical limitations are mainly carried out in modern western countries where the gender norms and associated social dynamics are different from India. One possible explanation may be that in the Indian patriarchal society, older men continue to get respect and health caring irrespective of their marital status as men have control over the resources and property rights. However, women deprived of such rights might cause the family members’ ignorant attitude toward older unmarried women as women are financially and socially dependent on their spouse if married and if unmarried then on their children or other relatives that further intensify the marriage role in the association between physical limitations and depressive symptoms.

A key strength of this empirical research is that the functional limitations of the Indian greying population are a significant factor in determining their level of depressive symptoms. Furthermore, a person’s marital status is a crucial protective mechanism against depressive symptoms. However, this study has some limitations as well. First, the result indicated the psychological distress of Indian older adults collectively and has not focused on either state or regional variations. Our study only focused on the association between the physical limitations and depressive symptoms. But evidence suggests that a potential bi-directional association may exist between physical limitations and depressive symptoms [14]. Another limitation is that our study analysis is confined to examine the moderating effect of marital status in an association of physical limitations and level of depressive symptoms in Indian older adults. However, the study has not examined how marital supports and conflicts converge with gender dimensions and moderate the effects of functional limitations on depressive symptoms [30, 43]. A critical subject for future research is exploring marital quality in the moderating processes studied in this research across different age groups, including younger ages.

## Conclusion

This empirical study identifies functional limitations as a stressor in late life. We have also inferred that the increased physical limitations among India’s older people preponderate the likelihood of depressive symptoms, and such transformation is visible among the cohort of females. Our observations underscore the necessity of considering the gender aspect in early ages and the late life of women. The lone woman, without spousal support, needed to get equal treatment and help from other family members and relatives, which may help to reduce her level of depression to some extent.

## Data Availability

The study utilises a secondary source of data that is freely available in the public domain through https://g2aging.org/

## Abbreviations

ADL: Activities of daily living
IADL: Instrumental activities of daily living
MD: Mobility difficulty
OR: Odds ratio
CI: Confidence interval
ST: Scheduled tribe
SC: Scheduled caste
OBC: Other backward class
